# Yoga-based group therapy for in-patients with schizophrenia spectrum disorders – a qualitative approach

**DOI:** 10.1192/j.eurpsy.2021.2135

**Published:** 2021-08-13

**Authors:** T. Schulze, E. Hahn, I.M. Hahne, N. Bergmann, M. Zierhut, T.M.T. Ta, M. Pijnenborg, K. Böge

**Affiliations:** 1 Faculty Of Behavioral And Social Sciences, University of Groningen, Groningen, Netherlands; 2 Department Of Psychiatry, Charité – Universitätsmedizin Berlin, Campus Benjamin Franklin, Berlin, Germany

**Keywords:** yoga-based group therapy, Schizophrenia spectrum disorders, qualitative approach, psychosis

## Abstract

**Introduction:**

Yoga may pose a promising complementary therapy in the multimodal treatment of schizophrenia spectrum disorders (SSD). However, to date, no studies have qualitatively examined the patients’ experience of practising Yoga.

**Objectives:**

This qualitative study aimed to assess the mechanisms and processes of Yoga-based group therapy (YBGT) for in-patients with SSD by exploring their subjective experiences.

**Methods:**

Twenty-five semi-structured interviews were conducted with in-patients with SSD after they participated in a YBGT session. Interviews were transcribed, coded by two independent researchers, and analysed using an inductive thematic approach. The research team collaboratively discussed emerging categories to reduce redundancy and form meaningful themes and subthemes.

**Results:**

The analysis revealed seven main themes. YBGT was perceived as feasible and focusing on individual adaptation, captured by the theme ‘inclusivity’. Nevertheless, participants encountered ‘challenges’; thus, physical limitations need to be considered. While practising together, participants experienced ‘interconnectedness’ and developed a ‘mindful stance’ as they accepted their limitations and adapted exercises with self-compassion. Following the flow of asanas required physical persistence, which ultimately led many participants to experience ‘confidence’ and ‘relaxation’. YBGT affected ‘symptom representation’ as heightened awareness led participants to notice impeding as well as improved symptoms.

**Conclusions:**

YBGT seemed to have various promising effects on in-patients with SSD. Future research should examine to what extent these effects can be sustained and how the mindful approach during YBGT can be transferred to areas outside the Yoga class. Furthermore, a randomised-controlled trial could investigate the effectiveness of a manualised YBGT.

**Disclosure:**

No significant relationships.

